# Supplementary figures and images for: The Advanced BRain Imaging on ageing and Memory (ABRIM) data collection: Study design, data processing, and rationale
Source: PLoS One. 2024 Jun 21;19(6):e0306006. doi: 10.1371/journal.pone.0306006 (PMC11192316; doi:10.1371/journal.pone.0306006)

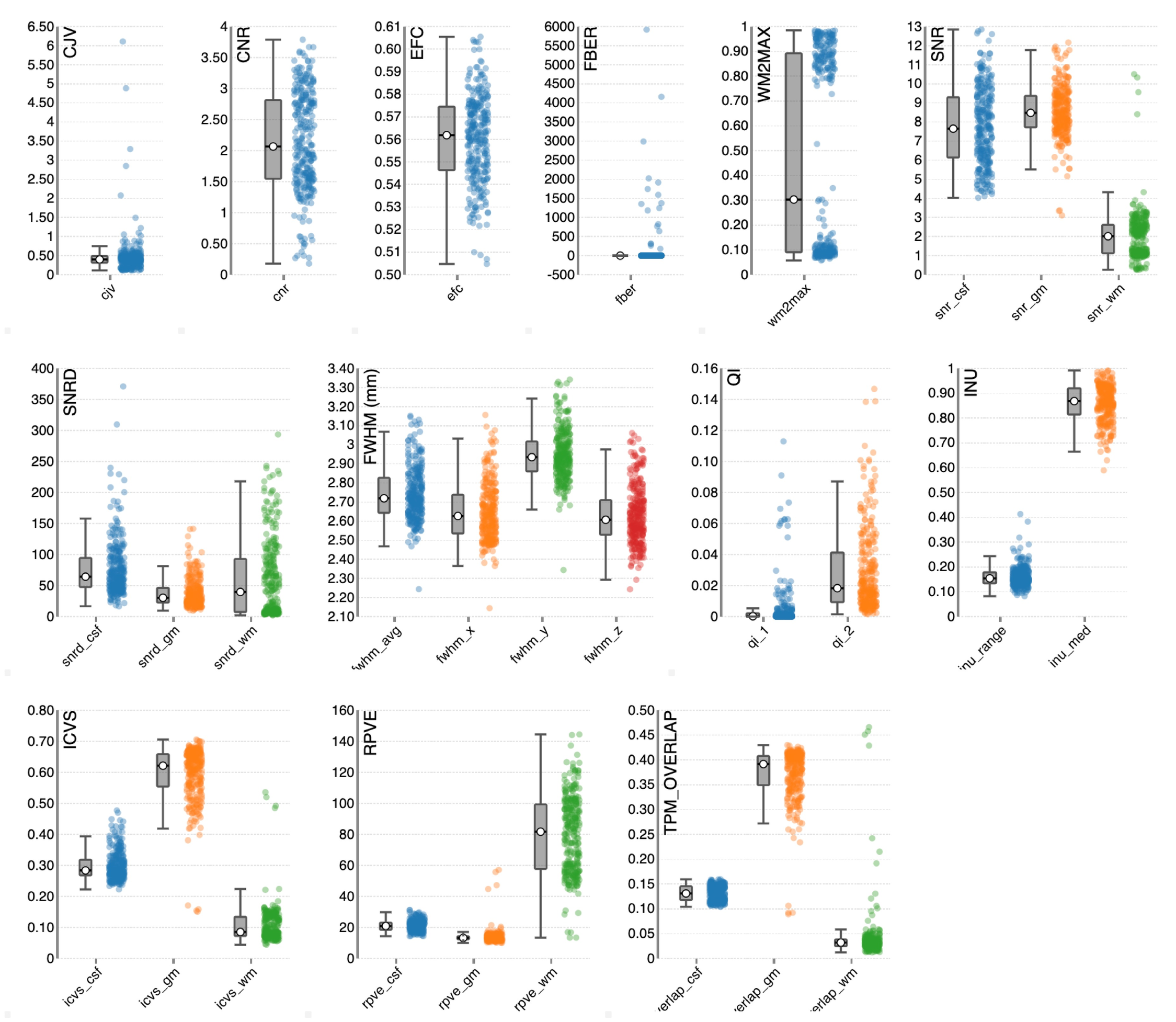

Supplement: S1 Fig — Group anatomical report of T2 scans in ABRIM as generated by the MRI Quality control tool (MRIQC). Contains separate strip-plots for different image quality metrics (IQMs). CJV, coefficient of joint variation; CNR, contrast-to-noise-ratio; EFC, entropy focus criterion; FBER, foreground-to-background energy ratio; WM2MAX, white-matter to maximum intensity ratio; SNR, signal-to-noise-ratio; SNRD, Dietrich’s signal-to-noise-ratio; FWHM (vox), full width half maximum in units of voxels; QI, quality index; INU, intensity non-uniformity; ICVS, intracranial volume fraction; RPVE, residual partial volume effect; TPM_OVERLAP, overlap of issue probability maps of the images and maps from the ICBM nonlinear-asymmetric 2009a template. (TIF) [file pone.0306006.s001.tif]

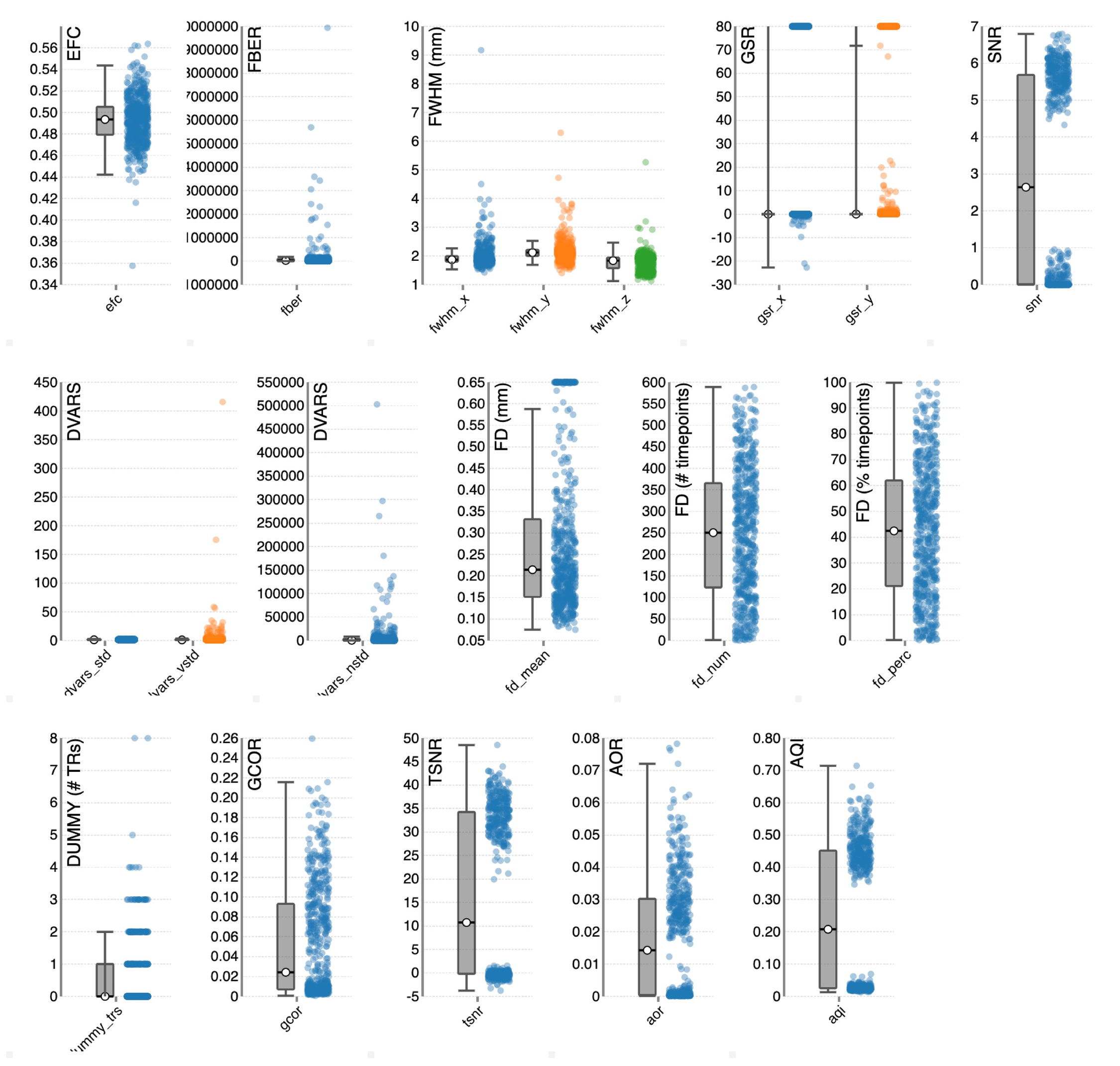

Supplement: S2 Fig — Group anatomical report of T2 scans in ABRIM as generated by the MRI Quality control tool (MRIQC). Contains separate strip-plots for different image quality metrics (IQMs). EFC, entropy focus criterion; FBER, foreground-to-background energy ratio; FWHM, full width half maximum in units of millimetres; GSR, ghost-to-signal ratio; SNR, signal-to-noise ratio; DVARS, index of rate of change of BOLD signal across the entire brain; FD, framewise displacement (number of timepoints and percentage of timepoints above threshold); DUMMY, number of dummy scans; GCOR, global time-series correlation; TSNR, temporal signal-to-noise ratio; AOR, AFNI’s outlier ratio; AQI, AFNI’s quality index. (TIF) [file pone.0306006.s002.tif]
