# Supplementary material for: The Advanced BRain Imaging on ageing and Memory (ABRIM) data collection: Study design, data processing, and rationale
Source: PLoS One. 2024 Jun 21;19(6):e0306006. doi: 10.1371/journal.pone.0306006 (PMC11192316; doi:10.1371/journal.pone.0306006)
Supplement: S1 Table — (PDF) [file pone.0306006.s003.pdf]

S1 Table. Overview variables in ABRIM relative to other databases.

Dataset name	Age range	Participants	Cognitive assessment	Actigraphy	T1-weighted	T2-weighted	MP2RAGE	DWI	T2* or GRE	Resting-state fMRI
Cambridge Centre for Ageing and Neuroscience (Cam-CAN)	18-87	~650	X		X	X		X		X
MPI-Leipzig Mind-Brain-Body	20-35; 59-77	227; 74	X			X	X	X	X	X
Neurocognitive aging data release	18-34; 60-89	181; 120	X		X					X
The Lifespan Human Connectome Project in Aging	36-100+	725	X		X	X		X		X
The National Institute of Health (NIMH) Intramural Healthy Volunteer Dataset	18-72	155	X		X	X		X	X	X
UK Biobank	40-70	~50,000	X	X	X			X	X	X
Whitehall II Imaging Sub-study	60-85	775	X		X	X		X		X
Lothian Birth Cohort 1936	±70	866	X		X	X	X	X	X	
The Advanced BRain Imaging on ageing and Memory	18-80	295	X	X	X	X	X	X	X	X
