# Supplementary material for: The Advanced BRain Imaging on ageing and Memory (ABRIM) data collection: Study design, data processing, and rationale
Source: PLoS One. 2024 Jun 21;19(6):e0306006. doi: 10.1371/journal.pone.0306006 (PMC11192316; doi:10.1371/journal.pone.0306006)
Supplement: S2 Table — Data on educational attainment were not available for n = 18 participants (6.4% of the total sample). For females and males, the respective numbers were n = 11 (7% of all females) and n = 7 (5.1% of all males). (PDF) [file pone.0306006.s004.pdf]

**S2 Table. Sample characteristics of full sample, per age decade and sex.**

| | | **Full sample** | **18-30 years** | **31-40 years** | **41-50 years** | **51-60 years** | **61-70 years** | **71-80 years** |
|---|---|---|---|---|---|---|---|---|
| **Full sample** | N | 295 | 46 | 45 | 48 | 51 | 53 | 52 |
| | Age, median (IQR) | 52 (36-66) | 25 (22-28) | 35 (33-38) | 47 (44-49) | 55 (53-57) | 65 (62.50-68) | 73 (72-76) |
| | Low education, N (%) | 36 (13%) | 3 (8.3%) | 4 (9.1%) | 4 (8.5%) | 6 (12.8%) | 9 (17.6%) | 10 (19.2%) |
| | Medium education, N (%) | 90 (32.6%) | 14 (38.9%) | 18 (40.9%) | 15 (31.9%) | 17 (36.2%) | 16 (31.4%) | 10 (19.2%) |
| | High education, N (%) | 151 (54.7%) | 19 (52.8%) | 22 (50%) | 28 (59.6%) | 24 (51.1%) | 26 (51%) | 32 (61.5%) |
| **Females** | N | 157 | 25 | 25 | 25 | 25 | 27 | 30 |
| | Age, median (IQR) | 52 (36.5-66.5) | 23 (23-26) | 36 (33-38) | 47 (44-49) | 55 (53-58) | 65 (62-68) | 73 (72-76) |
| | Low education, N (%) | 22 (15.1%) | 1 (5.3%) | 3 (12.5%) | 2 (8.3%) | 3 (13%) | 7 (26.9%) | 6 (20%) |
| | Medium education, N (%) | 49 (33.6%) | 8 (42.1%) | 10 (41.7%) | 7 (29.2%) | 10 (43.5%) | 7 (26.9) | 7 (23.3%) |
| | High education, N (%) | 75 (51.4%) | 10 (52.6%) | 11 (45.8%) | 15 (62.5%) | 10 (43.5%) | 12 (46.2%) | 17 (56.7%) |
| **Males** | N | 138 | 21 | 20 | 23 | 26 | 26 | 22 |
| | Age, median (IQR) | 52 (35.75-66) | 27 (24-29) | 33 (32-37.75) | 46 (43-48) | 55 (52.75-57) | 66 (63.50-68) | 73.50 (72-76) |
| | Low education, N (%) | 14 (10.7%) | 2 (11.8%) | 1 (5%) | 2 (8.7%) | 3 (12.5%) | 2 (8%) | 4 (18.2%) |

| | | | | | | | | |
|---|---|---|---|---|---|---|---|---|
| | Medium education, N (%) | 41 (31.3%) | 6 (35.3%) | 8 (40%) | 8 (34.8) | 7 (29.2%) | 9 (36%) | 3 (13.6%) |
| | High education, N (%) | 76 (58%) | 9 (52.9%) | 11 (55%) | 13 (56.5%) | 14 (58.3%) | 14 (56%) | 15 (68.2%) |

Data on educational attainment were not available for n = 18 participants (6.4% of the total sample). For females and males, the respective numbers were n = 11 (7% of all females) and n = 7 (5.1% of all males).
