# Supplementary material for: The Advanced BRain Imaging on ageing and Memory (ABRIM) data collection: Study design, data processing, and rationale
Source: PLoS One. 2024 Jun 21;19(6):e0306006. doi: 10.1371/journal.pone.0306006 (PMC11192316; doi:10.1371/journal.pone.0306006)
Supplement: S3 Table — Data on educational attainment was not available for n = 3 participants (2.8%). (PDF) [file pone.0306006.s005.pdf]

S3 Table. Sample characteristics of behavioural and cognitive assessment only participants.

	**Full sample**	**18-30 years**	**31-40 years**	**41-50 years**	**51-60 years**	**61-70 years**	**71-80 years**
N	108	37	7	14	22	20	8
Age, median (IQR)	48 (24-61)	22 (21-24.5)	32 (32-35)	47 (43-48)	55 (52-57.25)	64.5 (62-67)	72 (71.25-74.75)
Females, N (%)	64 (59.3%)	24 (64.9%)	5 (71.4%)	9 (64.3%)	9 (40.9%)	11 (55%)	6 (75%)
Low education, N (%)	5 (4.7%)	0 (0%)	1 (14.3%)	0 (0%)	0 (0%)	2 (10%)	2 (25%)
Medium education, N (%)	32 (31.1%)	16 (45.7%)	4 (57.1%)	5 (38.5%)	5 (22.7%)	2 (10%)	0 (0%)
High education, N (%)	68 (64.2%)	19 (54.3%)	2 (28.6%)	8 (61.5%)	17 (77.3%)	16 (80%)	6 (75%)

Data on educational attainment was not available for n = 3 participants (2.8%).
