# Supplementary material for: The Advanced BRain Imaging on ageing and Memory (ABRIM) data collection: Study design, data processing, and rationale
Source: PLoS One. 2024 Jun 21;19(6):e0306006. doi: 10.1371/journal.pone.0306006 (PMC11192316; doi:10.1371/journal.pone.0306006)
Supplement: S4 Table — Data on educational attainment was not available for n = 5 participants (4.2%). (PDF) [file pone.0306006.s006.pdf]

S4 Table. Characteristics of actigraphy sample.

	Full sample	18-30 years	31-40 years	41-50 years	51-60 years	61-70 years	71-80 years
N	120	13	11	16	26	28	26
Age, median (IQR)	57 (44.3-69)	26 (24-30)	33 (31-37)	45.50 (44-48.75)	54.50 (52-57)	65 (62-68)	73 (72-76)
Females, N (%)	68 (56.6%)	10 (76.9%)	7 (63.6%)	8 (50%)	12 (46.2%)	17 (60.7%)	14 (53.8%)
Low education, N (%)	14 (12.2%)	0 (0%)	1 (9.1%)	1 (6.3%)	1 (4.2%)	5 (19.2%)	6 (23.1%)
Medium education, N (%)	30 (26.1%)	6 (50%)	3 (27.3%)	4 (25%)	9 (37.5%)	4 (15.4%)	4 (15.4%)
High education, N (%)	71 (61.7%)	6 (50%)	7 (63.6%)	11 (68.8%)	14 (58.3%)	17 (65.4%)	16 (61.5%)

Data on educational attainment was not available for n = 5 participants (4.2%).
