# Supplementary material for: The Advanced BRain Imaging on ageing and Memory (ABRIM) data collection: Study design, data processing, and rationale
Source: PLoS One. 2024 Jun 21;19(6):e0306006. doi: 10.1371/journal.pone.0306006 (PMC11192316; doi:10.1371/journal.pone.0306006)
Supplement: S1 File — (PDF) [file pone.0306006.s007.pdf]

S1 File. English translation of memory strategy descriptions.

Many people use techniques or strategies to improve their recall of word combinations. Have you utilized any techniques or strategies to memorize the word pairs you heard? Could you provide a description of these strategies with as much detail as possible below?

[next page]

The following list consists of strategies or techniques that people can use to remember word combinations. Read the list of strategies and tick the applicable boxes to indicate which strategies you have employed to enhance your recall of the word pairs. Indicate all strategies that you have used, even if you already described them on the previous page.

Concentrate	☐
Repeat in your head	☐
Visualize (create imagesin your head)	☐
Making associations between the words of a pair	☐
Making a story containing words of a pair	☐
Making a sentence containing both words of a pair	☐
Visualize with yourself in a mental image	☐
Remember specific letters or syllables	☐
Memorize sounds of word combinations	☐
Something different, namely………………………………………	☐
